# Molecular Mechanism Underlying Hypoxic Preconditioning-Promoted Mitochondrial Translocation of DJ-1 in Hypoxia/Reoxygenation H9c2 Cells

**DOI:** 10.3390/molecules25010071

**Published:** 2019-12-24

**Authors:** Yi-Zhang Deng, Lin Xiao, Le Zhao, Le-Jia Qiu, Zhao-Xia Ma, Xing-Wang Xu, Hao-Yue Liu, Ting-Ting Zhou, Xue-Ying Wang, Lei Tang, He-Ping Chen

**Affiliations:** The Key Laboratory of Basic Pharmacology, School of Pharmaceutical Science, Nanchang University, Nanchang 330006, China; dyz1998@yahoo.com (Y.-Z.D.); xiaolin7752@163.com (L.X.); zle190801@163.com (L.Z.); qiulejia1997@126.com (L.-J.Q.); mzx1786696991@126.com (Z.-X.M.); xxw1798612667@126.com (X.-W.X.); LHY18770007086@126.com (H.-Y.L.); 17863205017@163.com (T.-T.Z.); wxy191714@126.com (X.-Y.W.); lvzuzu@hotmail.com (L.T.)

**Keywords:** DJ-1, mitochondria translocation, hypoxic preconditioning, Grp75, oxidative stress, hypoxia/reoxygenation

## Abstract

DJ-1 was recently reported to be involved in the cardioprotection of hypoxic preconditioning (HPC) against hypoxia/reoxygenation (H/R)-induced oxidative stress damage, by preserving mitochondrial complex I activity and, subsequently, inhibiting mitochondrial reactive oxygen species (ROS) generation. However, the molecular mechanism by which HPC enables mitochondrial translocation of DJ-1, which has no mitochondria-targeting sequence, to preserve mitochondrial complex I, is largely unknown. In this study, co-immunoprecipitation data showed that DJ-1 was associated with glucose-regulated protein 75 (Grp75), and this association was significantly enhanced after HPC. Immunofluorescence imaging and Western blot analysis showed that HPC substantially enhanced the translocation of DJ-1 from cytosol to mitochondria in H9c2 cells subjected to H/R, which was mimicked by DJ-1 overexpression induced by pFlag-DJ-1 transfection. Importantly, knockdown of Grp75 markedly reduced the mitochondrial translocation of DJ-1 induced by HPC and pFlag-DJ-1 transfection. Moreover, HPC promoted the association of DJ-1 with mitochondrial complex I subunits ND1 and NDUFA4, improved complex I activity, and inhibited mitochondria-derived ROS production and subsequent oxidative stress damage after H/R, which was also mimicked by pFlag-DJ-1 transfection. Intriguingly, these effects of HPC and pFlag-DJ-1 transfection were also prevented by Grp75 knockdown. In conclusion, these results indicated that HPC promotes the translocation of DJ-1 from cytosol to mitochondria in a Grp75-dependent manner and Grp75 is required for DJ-1-mediated protection of HPC on H/R-induced mitochondrial complex I defect and subsequent oxidative stress damage.

## 1. Introduction

Ischemic preconditioning is a powerful endogenous phenomenon protecting against acute myocardial ischemia/reperfusion (I/R) injury [1]. The protective effect is observed at several hours (early cardioprotection lasting several hours) and 12–24 h (late cardioprotection lasting several days) after ischemic preconditioning [2,3]. A wealth of information has been collected on the molecular mechanisms involved in ischemic preconditioning, but many aspects of the signaling pathways and of the end effectors of the cardioprotection remain unknown [2,4,5]. An intriguing and unresolved aspect is the involvement of DJ-1 in the genesis of preconditioning [6,7,8]. 

DJ-1, originally identified in 1997 as an oncogene product [9], is a ubiquitously expressed and highly conserved protein comprised of 189 amino acids and is associated with multiple biological functions, such as mitochondrial regulation, transcriptional regulation, molecular chaperone, signal transduction, and more [10,11]. Of most importance, dependable findings revealed that DJ-1 possesses an antioxidant activity and plays a role as a redox activated chaperone in cytoprotection under stimuli challenge [12,13]. Although it is not clear how DJ-1 is engaged in multiple biological processes, the ability of DJ-1 associating with various proteins could be one of the molecular bases supporting its various physiological functions [14]. 

Interestingly, we recently reported that DJ-1 is involved in the cardioprotection of hypoxic preconditioning (HPC) against hypoxia/reoxygenation (H/R)-induced oxidative stress damage as an endogenous protective protein, by preserving mitochondrial complex I activity and, subsequently, inhibiting mitochondrial reactive oxygen species (ROS) generation in H9c2 cells [15]. However, it is noteworthy that DJ-1 possesses no mitochondria-targeting sequence [11]. Therefore, how HPC induces the mitochondrial translocation of DJ-1 to preserve mitochondrial complex I is an important and interesting question for further exploration.

Glucose-regulated protein 75 (Grp75), also known as mtHsp70/PBP74/Mortalin, is the mitochondrial member of the heat shock protein family, an essential mitochondrial chaperone, and a prominent cytoprotective factor against various stresses [16]. The N-terminal region of Grp75 contains mitochondrial-targeting signal peptides, through which Grp75 can assist in the transport of numerous cytosolic proteins to the mitochondria, thus playing an important role in energy metabolism, stress protection, and mitochondrial homeostasis regulation [17,18]. More interestingly, it has been shown that both DJ-1 and Grp75 share common cytoprotective functions, including ROS regulation [19,20]. Moreover, there is evidence that DJ-1 is the binding partner of Grp75, and the binding of Grp75 to DJ-1 is involved in the management of mitochondrial oxidative stress [19,21]. Therefore, it was accordingly hypothesized that Grp75 could be an important regulatory factor in the mitochondrial translocation of DJ-1 by HPC. To test the hypothesis, we employed the cellular model of HPC and combined small interfering RNA (siRNA) technology with biochemical analysis, immunofluorescence images, and oxidative stress assay. Our data demonstrated that Grp75 could interact with DJ-1 to form a complex in response to HPC that is responsible for guiding DJ-1 to the mitochondria, in which DJ-1 could interact with ND1 and NDUFA4 subunits of mitochondrial complex I, preserves mitochondrial complex I activity, and inhibits mitochondria-derived ROS production and subsequent oxidative stress in the rat heart-derived H9c2 cells subjected to H/R. Collectively, these observations revealed a key molecular mechanism underlying the HPC-induced DJ-1 targeting to mitochondria.

## 2. Results

### 2.1. Effect of HPC on the Interaction between DJ-1 and Grp75 in H9c2 Cells Subjected to H/R 

**Figure 1:**
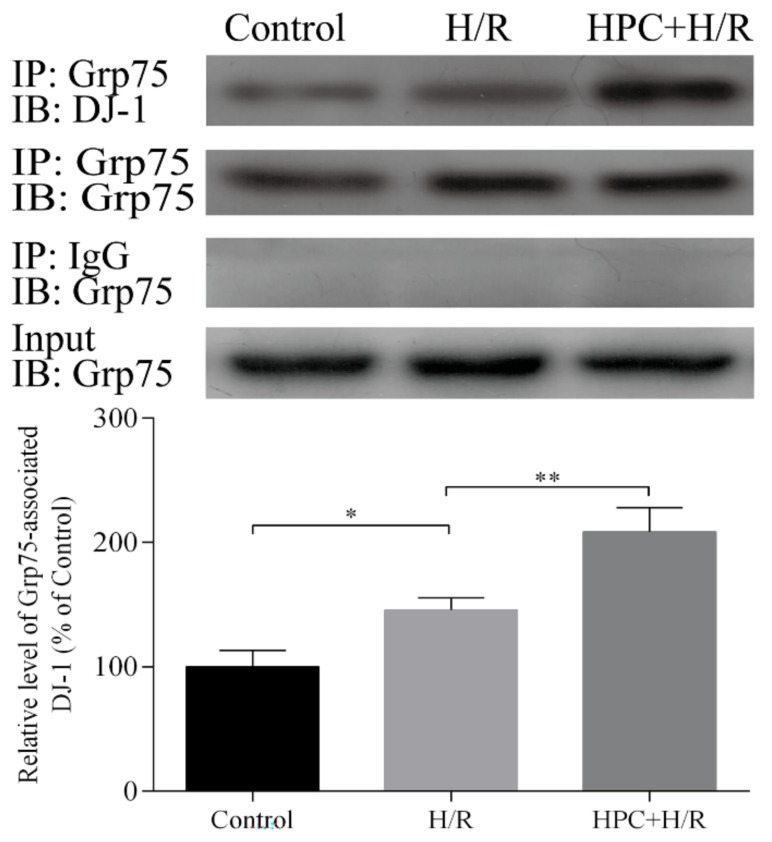
Effect of hypoxic preconditioning (HPC) on the interaction of DJ-1 and Grp75 in H9c2 cells subjected to hypoxia/reoxygenation (H/R). HPC was induced 24 h prior to H/R in H9c2 cells, and the interaction of DJ-1 and Grp75 was assessed by co-immunoprecipitation. Cell lysates were immunoprecipitated (IP) with IgG or anti-Grp75 antibody and immunoblot (IB) analysis was performed with anti-DJ-1 or anti-Grp75. Total protein extracts (prior to IP) were used as control (Input) and IgG-precipitated template served as specificity control. Representative blots from three independent experiments and bar graphs from their densitometric analyses are shown. The data are presented as mean ± SD. * *p* < 0.05, ** *p* < 0.01 as indicated on the figure.

Grp75 is an important molecular chaperone, which can associate with certain proteins lacking mitochondrial-targeting sequences to transport them into the mitochondria. To determine the role of Grp75 in the mitochondrial translocation of DJ-1 by HPC in H/R-treated H9c2 cardiomyocytes, the effect of HPC on the interaction of DJ-1 and Grp75 was first examined. The co-immunoprecipitation results showed that there could be a low level of interaction between DJ-1 and Grp75 under normal conditions, which was increased after H/R treatment. Interestingly, HPC 24 h prior to H/R further increased the interaction between DJ-1 and Grp75 in H9c2 cardiomyocytes (Figure 1). These results suggest that Grp75 could participate in DJ-1 mitochondrial translocation by HPC through associating with it in H/R-treated H9c2 cardiomyocytes.

### 2.2. Effect of Grp75 Knockdown on HPC-Promoted Mitochondrial Translocation of DJ-1 in H9c2 Cells Subjected to H/R

**Figure 2:**
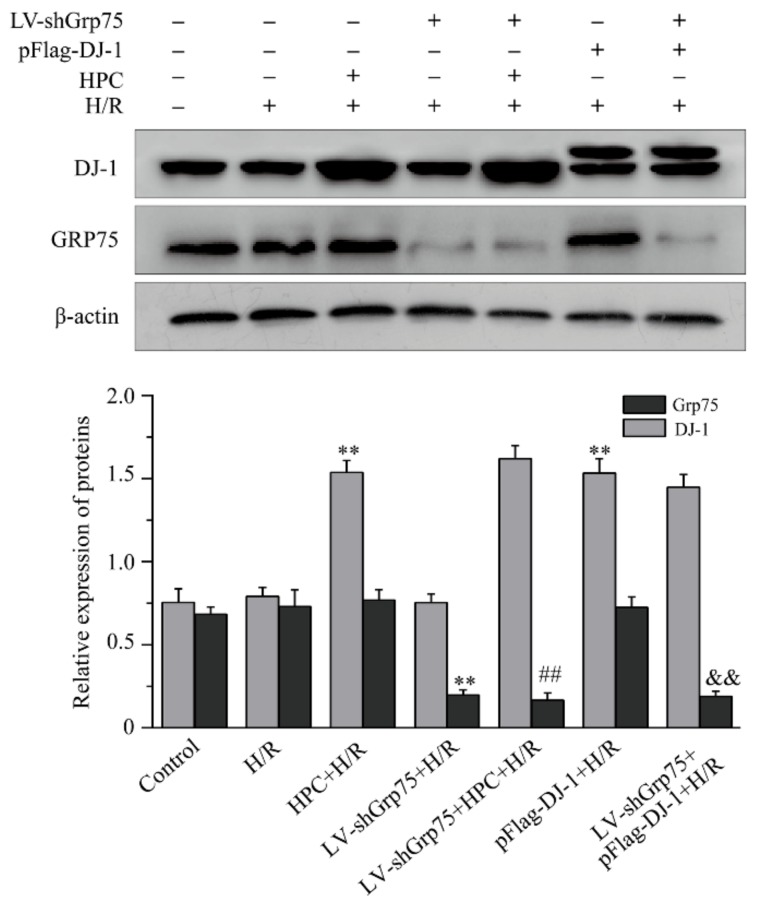
Western blot analysis of DJ-1 and Grp75 proteins expression in different treatment H9c2 cells. H9c2 cells were infected by lentiviral (LV) vectors LV-shGrp75 for 72 h and then subjected to hypoxic preconditioning (HPC) 24 h prior to hypoxia/reoxygenation (H/R) or transfected with pFlag-DJ-1 for 48 h followed by H/R. The expressions of DJ-1 and Grp75 proteins were determined by Western blot with the densitometric analysis normalized by β-actin. A representative blot of each experiment is shown with the densitometric analysis corresponding to the mean ± SD of three independent experiments. ** p < 0.01 vs. H/R group; ^##^ *p* < 0.01 vs. HPC + H/R group; *^&&^ p* < 0.01 vs. pFlag-DJ-1 + H/R group.

**Figure 3:**
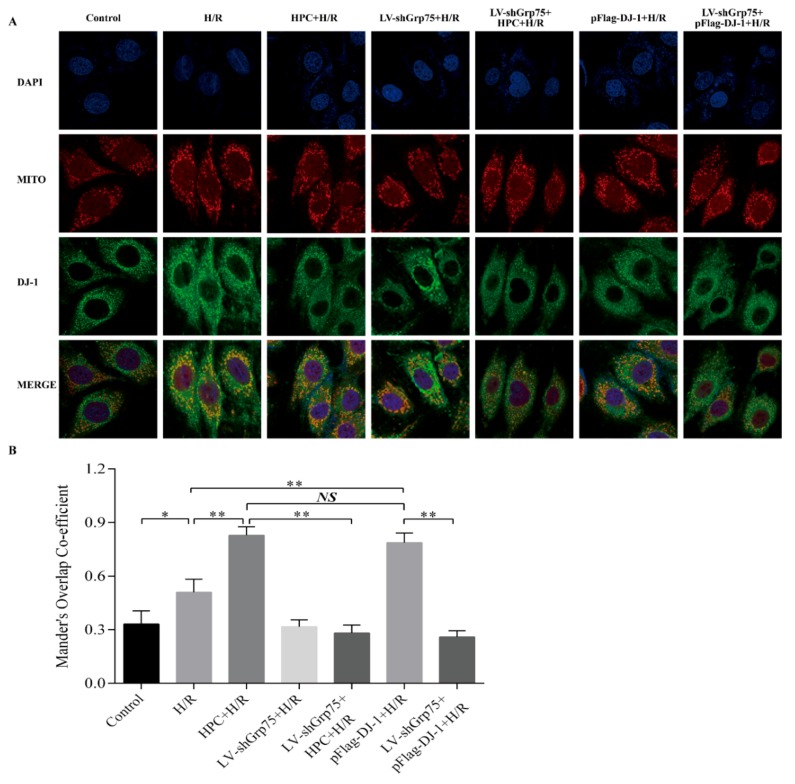
Effect of Grp75 knockdown on hypoxic preconditioning (HPC)-promoted the mitochondrial localization of DJ-1 in H9c2 cells subjected to hypoxia/reoxygenation (H/R). H9c2 cells were infected by lentiviral (LV) vectors LV-shGrp75 for 72 h and then subjected to HPC 24 h prior to H/R, or transfected with pFlag-DJ-1 for 48 h followed by H/R. Subsequently, the mitochondrial localization of DJ-1 was detected by immunofluorescence using an anti-DJ-1 antibody (Green), mitochondrial staining with MitoTracker (MITO) (Red), and nuclear staining with DAPI (Blue). (**A**) Representative fluorescent images from three separate experiments. (**B**) Quantification of the degree of colocalization between DJ-1 and MitoTracker. The degree of colocalization was assessed with Mander’s overlap coefficient obtained by using the plugin Wright Cell Imaging Facility (WCIF)-ImageJ for colocalization analysis for ImageJ NIH software. Data represent mean ± SD. * *p* < 0.05, ** *p* < 0.01 as indicated on the figure.

**Figure 4:**
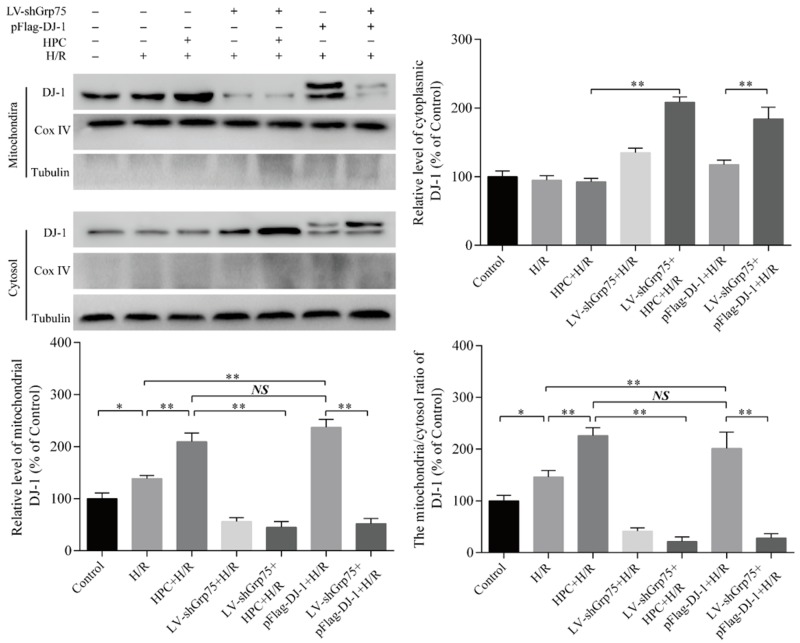
Effect of inhibiting Grp75 expression on hypoxic preconditioning (HPC)-induced DJ-1 translocation from cytosol to mitochondria in H9c2 cells subjected to hypoxia/reoxygenation (H/R). H9c2 cells were infected by lentiviral (LV) vectors LV-shGrp75 for 72 h and then subjected to HPC 24 h prior to H/R or transfected with pFlag-DJ-1 for 48 h followed by H/R. Subsequently, DJ-1 mitochondrial translocation was analyzed by Western blot. Cox IV and Tubulin served as loading and purity controls for mitochondrial and cytoplasmic fraction, respectively. The mitochondria:cytosol ratio of DJ-1 was used as indexes of DJ-1 translocation from cytosol to mitochondria. Representative Western blots from three independent experiments and bar graphs from their densitometric analyses are shown. Values are mean ± SD. * *p* < 0.05, ** *p* < 0.01 as indicated on the figure. NS indicates no significance.

Subsequently, to further clarify the necessary role of Grp75 in DJ-1’s translocation to mitochondria by HPC, the effect of Grp75 knockdown on the mitochondrial translocation of DJ-1 by HPC was observed in H9c2 cells subjected to H/R. Data shown in Figure 2 again demonstrated that HPC up-regulated the expression of DJ-1 protein, but had no significant effect on Grp75 expression. Moreover, the infection of LV-shGrp75 led to approximately 73–82% reduction of endogenous Grp75 in H/R-treated H9c2 cells but did not affect the total level of DJ-1 expression (Figure 2). Interestingly, the results of both immunofluorescence microscopy (Figure 3) and cell fractionation experiments (Figure 4) showed that HPC obviously promoted DJ-1 translocation, from cytoplasm to mitochondria, in H9c2 cells subjected to H/R, which was mimicked by DJ-1 overexpression induced by pFlag-DJ-1 transfection. More importantly, Grp75 knockdown apparently inhibited the mitochondrial translocation of DJ-1 by HPC or pFlag-DJ-1 transfection in H9c2 cells subjected to H/R (Figure 3 and Figure 4). Overall, these results indicated that Grp75 is required for HPC-promoted translocation of DJ-1 from the cytosol to mitochondria in H9c2 cells subjected to H/R.

### 2.3. Effect of Grp75 Knockdown on DJ-1-Mediated Protection of Mitochondrial Complex I by HPC in H9c2 Cells Subjected to H/R

**Figure 5:**
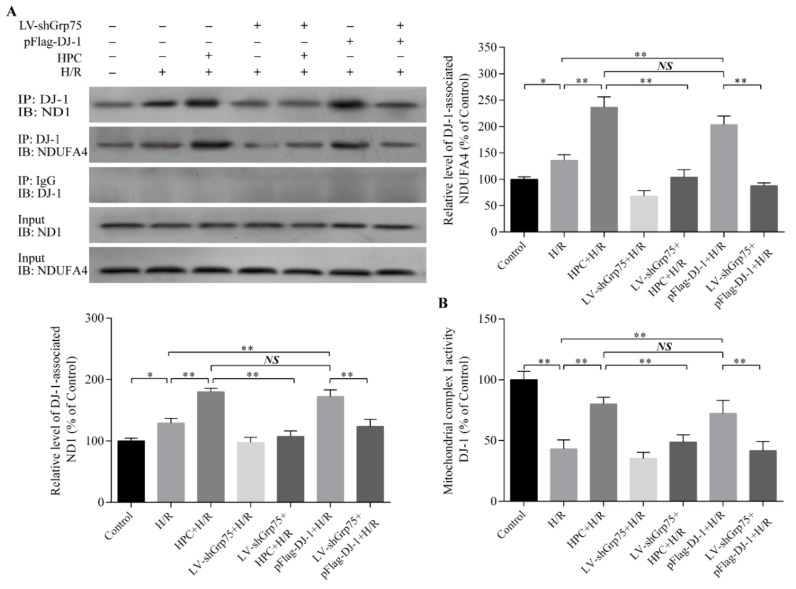
Effect of Grp75 knockdown on the association of DJ-1 with ND1 and NDUFA4 subunits and the protection of mitochondrial complex I activity by hypoxic preconditioning (HPC) in H9c2 cells subjected to hypoxia/reoxygenation (H/R). H9c2 cells were infected by lentiviral (LV) vectors LV-shGrp75 for 72 h and then subjected to HPC 24 h prior to H/R or transfected with pFlag-DJ-1 for 48 h followed by H/R. Subsequently, (**A**) the association of DJ-1 to NDUFA4 and ND1 was assessed by coimmunoprecipitation. Mitochondrial lysates were immunoprecipitated (IP) with anti-DJ-1 antibody, and immunoblot (IB) analysis was performed with anti-NDUFA4 or anti-ND1. Total protein extracts (prior to IP) were used as control (Input) and IgG-precipitated template served as specificity control. Representative blots from three independent experiments and bar graphs from their densitometric analyses are shown. The data are presented as mean ± SD. * *p* < 0.05, ** *p* < 0.01 as indicated on the figure. (**B**) The enzyme activity of mitochondrial complex I was determined as described in Materials and methods section. Each value represents the mean ± SD of three independent experiments. ** *p* < 0.01 as indicated on the figure. NS indicates no significance.

Our previous work suggested that HPC could protect the mitochondrial complex I activity and inhibit mitochondrial ROS production and subsequent oxidative stress in H/R cardiomyocytes through upregulating DJ-1 expression and promoting DJ-1 mitochondrial translocation. Therefore, we next observed whether the knockdown of Grp75 expression, and thereby inhibition of DJ-1 mitochondrial translocation, could affect the association of DJ-1 with ND1 and NDUFA4 subunits of mitochondrial complex I, and the subsequent protection of complex I by HPC in H/R-treated H9c2 cells. As shown in Figure 5, HPC enhanced the possible interaction between DJ-1 with ND1 and NDUFA4 (Figure 5A) accompanied by an increase in mitochondrial complex I activity (Figure 5B) relative to the H/R-treated cells alone. Similarly, the above effects of HPC were reproduced in pFlag-DJ-1-transfected H9c2 cells. Remarkably, when Grp75 expression was specifically knocked down by LV-shGrp75 infection, the above effects of both HPC and pFlag-DJ-1 transfection were significantly attenuated. These data suggested that Grp75 could be required for DJ-1-mediated protection of HPC on mitochondrial complex I activity in H9c2 cardiomyocytes subjected to H/R.

### 2.4. Effects of Grp75 Knockdown on DJ-1-Mediated Inhibition of Mitochondrial ROS Generation by HPC in H9c2 Cells Subjected to H/R

**Figure 6:**
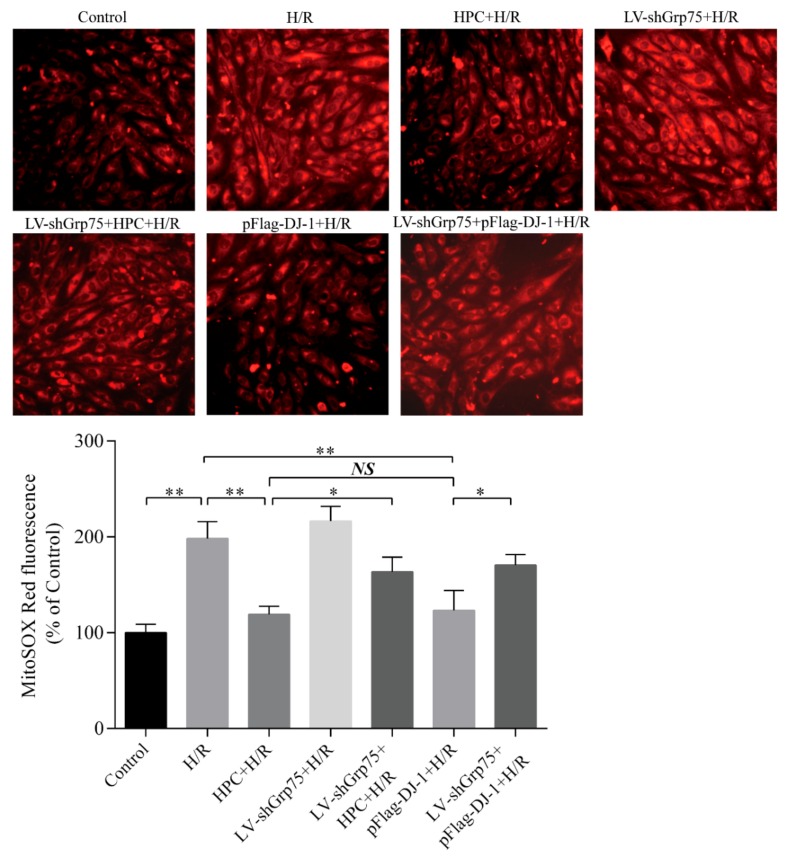
Effects of Grp75 knockdown on DJ-1-mediated inhibition of mitochondrial ROS generation by hypoxic preconditioning (HPC) in H9c2 cells subjected to hypoxia/reoxygenation (H/R). H9c2 cells were infected by lentiviral (LV) vectors LV-shGrp75 for 72 h and then subjected to HPC 24 h prior to H/R or transfected with pFlag-DJ-1 for 48 h followed by H/R. Subsequently, the mitochondrial SOX generation was measured by a MitoSOX Red fluorescent staining. (**A**) Representative fluorescence images of mitochondrial ROS observed with MitoSOX Red probe by fluorescent microscope (200×). (**B**) Quantitative analyses of MitoSOX Red fluorescence intensity in different treatment cells using a fluorescent plate reader. Each value represents the mean ± SD of three independent experiments. * *p* < 0.05, ** *p* < 0.01 as indicated on the figure. NS indicates no significance.

Accordingly, the present study next determined whether the knockdown of Grp75 expression and thereby inhibition of DJ-1 mitochondrial translocation could affect the inhibitory effect of HPC on mitochondrial ROS generation in H/R-treated H9c2 cells. Expectedly, the red fluorescence intensity of MitoSOX was increased 2.04-fold in the H/R cells compared to the control cells (Figure 6), indicating an increase in mitochondrial ROS generation after H/R. However, HPC significantly attenuated H/R–induced mitochondrial ROS accumulation, as evidenced by a lower MitoSOX fluorescence intensity, which was mimicked by DJ-1 overexpression induced by pFlag-DJ-1 transfection. Notably, the inhibitory effects of HPC and pFlag-DJ-1 transfection were antagonized by Grp75 knockdown (Figure 6). These results indicated that Grp75 is required for DJ-1–mediated the inhibitory effect of HPC on mitochondrial ROS release in H/R-treated H9c2 cells.

### 2.5. Effect of Grp75 Knockdown on DJ-1-Mediated Suppression of Oxidative Stress by HPC in H/R-Treated H9c2 Cells

**Figure 7:**
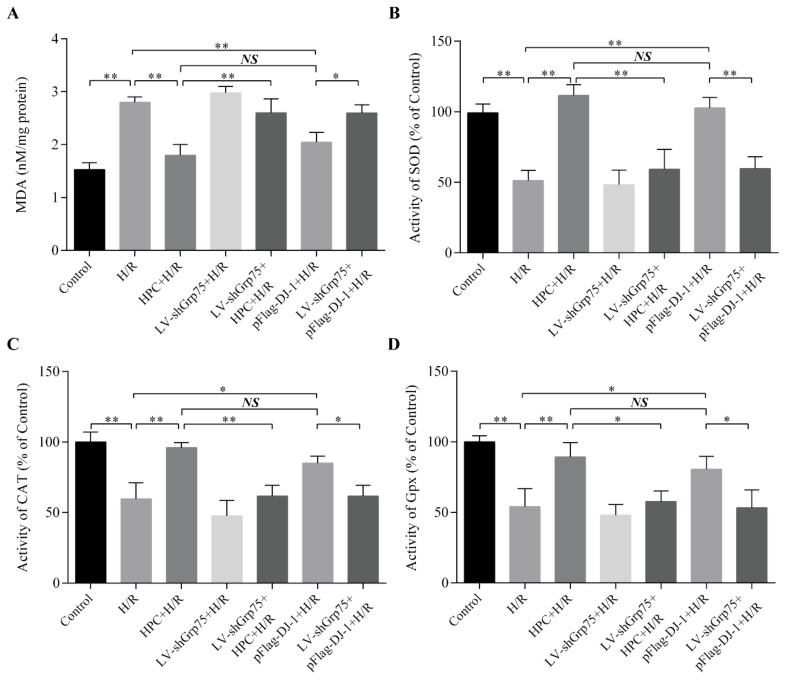
Effects of Grp75 knockdown on DJ-1-mediated inhibition of oxidative stress by hypoxic preconditioning (HPC) in H9c2 cells undergoing hypoxia/reoxygenation (H/R). H9c2 cells were infected by lentiviral (LV) vectors LV-shGrp75 for 72 h and then subjected to HPC 24 h prior to H/R or transfected with pFlag-DJ-1 for 48 h followed by H/R. Subsequently, oxidative stress was monitored by measuring the MDA content (**A**) and total activities of antioxidant enzyme superoxide dismutase (SOD) (**B**), catalase (CAT) (**C**), and glutathione peroxidase (GPx) (**D**), as described in the Materials and Methods section. Each value represents the mean ± SD of three independent experiments. * *p* < 0.05, ** *p* < 0.01 as indicated on the figure. NS indicates no significance.

Naturally, it was next investigated whether knockdown of Grp75 expression, and thereby inhibition of DJ-1 mitochondrial translocation, could affect DJ-1-mediated inhibitory effect of HPC on H/R-induced oxidative stress. Oxidative stress was monitored by measuring the malondialdehyde (MDA) content and total activities of antioxidant enzymes (superoxide dismutase (SOD), catalase (CAT), and glutathione peroxidase (GPx)). As presented in Figure 7, H9c2 cells exposed to H/R displayed an obvious increase in intracellular MDA content (Figure 7A), and a marked decrease in SOD (Figure 7B), CAT (Figure 7C), and GPx (Figure 7D) activities compared with the control group, implying that oxidative stress occurred after H/R. In contrast, HPC significantly suppressed oxidative stress caused by H/R, as demonstrated by higher SOD, CAT, and GPx activities, and lower MDA content than that observed for the H/R group. Moreover, the effects of HPC were also mimicked by pFlag-DJ-1 transfection-induced DJ-1 overexpression. Importantly, when H9c2 cells were infected with LV-shGrp75 for 72 h prior to HPC or pFlag-DJ-1 transfection, the inhibitory effects of HPC and pFlag-DJ-1 transfection on H/R-induced oxidative stress were significantly attenuated. These results suggested that Grp75 is required for DJ-1-mediated inhibitory effect of HPC on H/R-induced oxidative stress in H9c2 cardiomyocytes.

### 2.6. Effect of Grp75 Knockdown on DJ-1-Mediated Cardioprotection of HPC Against H/R Injury in H9c2 Cells 

**Figure 8:**
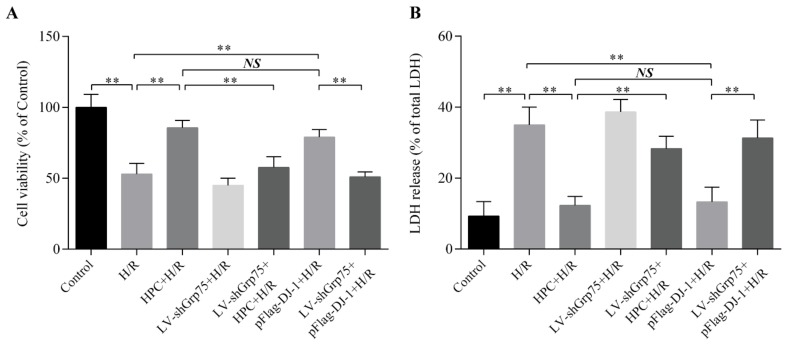
Effects of Grp75 knockdown on DJ-1-mediated cardioprotection of hypoxic preconditioning (HPC) in H9c2 cells undergoing hypoxia/reoxygenation (H/R). H9c2 cells were infected by lentiviral (LV) vectors LV-shGrp75 for 72 h and then subjected to HPC 24 h prior to H/R or transfected with pFlag-DJ-1 for 48 h followed by H/R. Subsequently, the cellular damage was analyzed by measuring cell viability (**A**) and lactate dehydrogenase (LDH) release (**B**), as described in Materials and Methods section. Each value represents the mean ± SD of three independent experiments. ** *p* < 0.01 as indicated on the figure. NS indicates no significance.

Finally, it was analyzed whether knockdown of Grp75 expression, and thereby inhibition of DJ-1 mitochondrial translocation, could affect the cardioprotective effect of HPC against H/R injury. Cellular damage was analyzed by measuring cell viability and LDH release. As presented in Figure 8, H/R significantly reduced cell viability (Figure 8A) and increased LDH release (Figure 8B). However, HPC significantly attenuated the LDH release and viability loss induced by H/R, again indicating that HPC can elicit the cardioprotection against H/R injury. Likewise, the cardioprotection of HPC was mimicked by pFlag-DJ-1 transfection. More importantly, Grp75 knockdown by LV-shGrp75 also abrogated the cardioprotection of HPC and pFlag-DJ-1 transfection. These results clearly indicated that Grp75 is required for DJ-1-mediated cardioprotection of HPC against H/R injury.

## 3. Discussion

The key finding of the present study was that HPC enhances the translocation of DJ-1 from cytosol to mitochondria in a Grp75-dependent manner in H9c2 cells subjected to H/R. Moreover, it was further demonstrated that Grp75 is critical in DJ-1-mediated protection of HPC on H/R-induced mitochondrial complex I defect and oxidative stress injury, probably by associating with DJ-1 and subsequently guiding its mitochondrial translocation. To our knowledge, this study is the first description that Grp75 could associate with DJ-1, acting as a molecular chaperone to transport it into the mitochondria under HPC conditions and suggests a key molecular mechanism underlying HPC-promoted mitochondrial translocation of DJ-1 in H9c2 cells subjected to H/R.

DJ-1 is a small, ubiquitously-expressed cytoprotective protein [22]. Subcellular localization studies have shown DJ-1 is mainly localized in the cytoplasm under basal conditions, but under oxidative stress it can be translocated to the mitochondria to exert cytoprotection function against oxidative stress [23,24]. Despite the functional evidence, little is known about the regulation of DJ-1 subcellular localization. 

The important role of DJ-1 as a potential mediator of HPC has been investigated in ours previous published experimental works, and studies have shown that DJ-1 is implicated in the cardioprotection of HPC against H/R-induced oxidative stress damage [6,8,25]. Interestingly, our recent studies have demonstrated that DJ-1-mediated cardioprotection is related to preserving mitochondrial complex I activity and subsequently inhibiting mitochondrial ROS generation [15]. This is again confirmed by the present study. Importantly, the present study provided further evidence that HPC enhances the translocation of DJ-1 from cytosol to mitochondria in H9c2 cells subjected to H/R. It is well known that many proteins translocated into mitochondria possess the N-terminal mitochondrial targeting sequence that is recognized by the mitochondrial import machinery [26]. However, no mitochondrial targeting sequence in DJ-1 has been found [11]. Therefore, how HPC induces the mitochondrial translocation of DJ-1 is an important and interesting question for further exploration. Accordingly, in the present study, we focused our attention on investigating the underlying mechanisms in H9c2 cells.

Grp75 has been shown to bind directly or associate with certain proteins lacking a mitochondrial-targeting sequence, thereby mediating their mitochondrial translocation by acting as an essential component of the translocational machinery in the inner mitochondrial membrane complex [16,27]. Interestingly, it has been shown that both DJ-1 and Grp75 share common cytoprotective functions. Moreover, it was reported that DJ-1 is the binding partner of Grp75, and the binding guards against mitochondrial oxidative stress [19,21,28]. Hence, it is reasonable to hypothesize that Grp75 may be involved in enhanced mitochondrial translocation of DJ-1 by HPC in H9c2 cells subjected to H/R. In support of this hypothesis, our co-immunoprecipitation experiments demonstrated that Grp75 could be associated with DJ-1 under normal conditions as well as following HPC. This association was enhanced following HPC, indicating that increased association of DJ-1 with Grp75 may likely contribute to the enhanced mitochondrial import of DJ-1. More importantly, our data further showed that reduced expression of Grp75 by siRNA prevented the HPC-induced increase in DJ-1 mitochondrial translocation, indicating that Grp75 is required for HPC-promoted translocation of DJ-1 from the cytosol to mitochondria in H9c2 cells subjected to H/R. However, whether Grp75 interacts directly with DJ-1 remains to be further clarified and the precise mechanism by which Grp75 assists in DJ-1 mitochondrial translocation also needs further investigation. Furthermore, in this regard, it has to be noted that more than 50% of the mitochondrial proteins do not use the classical import pathway that requires the recognition of a specific sequence [29]. Many proteins, such as NF-kB and AP-1, have been detected in mitochondria despite lacking a canonical mitochondria-targeting sequence, indicating the existence of still unknown mechanisms of intracellular trafficking [30].

Several lines of evidence suggested that DJ-1 plays a major role in maintenance of mitochondrial complex I activity and improvement of mitochondrial function [31,32]. It has now been recognized that ND1 and NDUFA4, as the important subunits of mitochondrial complex I, are the key sites regulating the activity of mitochondrial complex I and the generation of mitochondrial ROS [33]. Experimental evidence has shown that dysfunction of ND1 and NDUFA4 impairs the assembly and activity of mitochondrial complex I, which increases the electron leakage by blocking the electron transfer and leads to mitochondrial ROS overproduction [34,35]. Of note, Hayashi et al. reported that DJ-1 can associate with NDUFA4 and ND1 to contribute to the assembly and activity maintenance of complex I [31]. By analogy, the present study demonstrated that HPC promoted the association of DJ-1 with ND1 and NDUFA4 accompanied by improvement of complex I activity and suppression of mitochondrial ROS production and subsequent oxidative stress damage after H/R, which were also mimicked by DJ-1 overexpression induced by pFlag-DJ-1 transfection. These observations further support the potential role of DJ-1 in HPC-induced complex I preservation and subsequent oxidative stress attenuation probably through binding to ND1 and NDFU4. Most intriguingly, the knockdown of Grp75 expression, and thereby inhibition of DJ-1 mitochondrial translocation, alleviated the above effects induced by HPC and DJ-1 overexpression. The data strongly supported that Grp75 is an important regulatory factor in DJ-1-mediated protection of HPC on H/R-induced mitochondrial complex I defect and oxidative stress injury probably by associating with DJ-1, thereby mediating its mitochondrial translocation. Nonetheless, of note, in this study, it is insufficient to determine that there is a direct interaction of DJ-1 with ND1 and NDUFA4 only by immunoprecipitation experiment, and can not also be concluded that DJ-1-mediated inhibition of HPC on mitochondrial ROS generation is due directly to the binding of DJ-1 to the ND1 and NDUFA4 subunits. Therefore, whether DJ-1 mediates protection of HPC in H9c2 cells subjected to H/R by direct binding to NDUFA4 and ND1, and thereby inhibiting mitochondrial ROS generation after translocation to mitochondria in a Grp75-dependent manner, remains to be further confirmed. Additionally, there is considerable evidence that DJ-1 plays an important role in inhibition of mPTP opening [7,36] and mitophagy stimulation [37,38]. Therefore, whether DJ-1-mediated cardioprotection of HPC is related to inhibition of mPTP opening and mitophagy stimulation is also an important and interesting question for future research.

In conclusion, the present study provides an understanding of the molecular mechanism underlying HPC-promoted mitochondrial translocation of DJ-1, which increase our knowledge about DJ-1-mediated cardioprotection of HPC against H/R-induced oxidative stress injury, and may lead to identification of potential therapeutic targets for ischemic heart diseases.

## 4. Materials and Methods

### 4.1. Chemicals and Reagents

Dulbecco’s modified Eagles medium (DMEM) and fetal bovine serum (FBS) were obtained from Gibcol (Grand Island, NY, USA). MitoSOX™ Red Mitochondrial Superoxide Indicator was purchased from Molecular Probes Inc. (Eugene, OR, USA). All of the primary antibodies were obtained from Abcam (Cambridge, MA, USA). MDA, SOD, CAT, and GPx assay kits were obtained from Jiancheng Bioengineering Institute (Jiancheng, Nanjing, China). Other reagents were all purchased from Sigma-Aldrich (St. Louis, MO, USA), unless otherwise stated.

### 4.2. Cell Culture

Rat embryonic cardiomyoblast-derived H9c2 cells were obtained from the American Type Culture Collection (Manassas, VA, USA) and cultured in DMEM supplemented with 2 mM L-glutamine, 10% (*v/v*) FBS, and penicillin (100 U/mL)/streptomycin (100 μg/mL). Cells were maintained in a humidified incubator (Sanyo, Osaka, Japan) (95% air/5% CO_2_ at 37 °C) until 70–80% confluent prior to various experiments.

### 4.3. Cell Transfection

The plasmid pFlag-DJ-1 was constructed and stored by our laboratory [39] and transfected into H9c2 cells using Lipofectamine 2000 reagent according to the instructions of the manufacturer. Briefly, cells were seeded into six-well plates at a density of 1 × 10^5^ cells/well and transfected with 20 µg of plasmids/well. After a 48 h incubation, the cells were harvested for the subsequent experiment. Grp75-knockdown lentiviral vector (LV-shGrp75) was commercially constructed by the GeneChem Corporation (Shanghai, China), which was infected into H9c2 cells at the multiplicity of infection of 100 pfu/cell following the manufacturer’s instruction. The empty lentiviral vectors were used as negative control. After infection for 72 h, the cells were then harvested for further experiments.

### 4.4. Establishment of Cellular Models of H/R and HPC

HPC and H/R models were constructed as described previously with minor modifications [40,41,42]. Briefly, to induce H/R injury, H9c2 cells were initially equilibrated for 30 min with normal Tyrode solution (125 mM NaCl, 2.6 mM KCl, 1.2 mM KH_2_PO_4_, 1.2 mM MgSO_4_, 1.0 mM CaCl_2_, 25 mM HEPES, and 25 mM glucose, pH 7.4) at 37 °C. Subsequently, the cells were incubated in glucose-free Tyrode solution (pH 6.8, equilibrated with a gas mixture of 95% N_2_ and 5% CO_2_ for 15 min) at 37 °C for 3 h in a hypoxia chamber (Thermo scientific, Waltham, MA, USA) with a compact oxygen controller, where oxygen concentration was maintained at 1% by injecting a gas mixture of 95% N_2_ and 5% CO_2_. Following hypoxic exposure, the cells were incubated with normal Tyrode solution and restored to normoxic condition (95% air and 5% CO_2_ at 37 °C) for reoxygenation for another 2 h. Hypoxic preconditioning was carried out by exposing cells to 10 min of hypoxia and 30 min of reoxygenation 24 h prior to H/R. For control, H9c2 cells were incubated in normal Tyrode solution during the entire experimental period.

### 4.5. Immunofluorescence Microscopy

H9c2 cells grown on coverslips were fixed in 4% paraformaldehyde in phosphate-buffered saline (PBS) for 15 min, washed with PBS three times, and permeabilized with 0.5% Triton X-100 in PBS for 10 min. After washing with PBS again and blocking with 5% bovine serum albumin (BSA) for 1 h, the cells were incubated with the indicated primary antibodies for 2 h followed by incubation with fluorescein isothiocyanate (FITC)-conjugated secondary antibodies for 1 h at room temperature. Nuclei were identified by 10 μM DAPI staining for 10 min. Mitochondria were identified by 100 nM MitoTracker Red (Molecular Probes, Eugene, OR, USA) staining for 30 min at 37 °C before the cells were fixed. Images were acquired using a confocal laser-scanning microscope (Axiovert 200, Zeiss).

### 4.6. Western Blot Analysis

Whole proteins were obtained from H9c2 cardiomyocytes with the Protein Extraction Kit (Sigma-Aldrich, St. Louis, MI, USA) by following manufacturer’s protocols, whereas cytosolic and mitochondrial proteins were obtained with the Mitochondria/Cytosol Fractionation Kit (Pierce, Rockford, IL, USA). The protein concentration was quantified by the Lowry method using a Bio-Rad DC protein assay kit II. Equal amounts of proteins were separated by 12% SDS-PAGE gel and transferred to polyvinylidene difluoride membranes (Millipore, Boston, MA, USA). After blocking nonspecific binding sites for 1 h with 5% non-fat milk, the membranes were then probed for 2 h at room temperature or at 4 °C overnight with primary antibodies (1:1000 dilution). After washing, the membranes were incubated with appropriate secondary antibodies (1:3000) and detected by an enhanced chemiluminescence kit (Millipore, Boston, MA, USA). The band intensities were analyzed using Quantity One^®^ image analysis software version 4.62 (Bio-Rad Laboratories, Inc.). To confirm the purity of mitochondrial-cytoplasmic fractionation, the extracts were probed with cytosol-specific anti-tubulin (1:1000) and mitochondria-specific anti-Cox IV (1:1000) antibodies.

### 4.7. Co-Immunoprecipitation Assays

After experimental treatments, the cells were harvested and lysed in immunoprecipitation (IP) lysis buffer (50 mM Tris HCl, 150 mM NaCl, 1 mM ethylenediaminetetraacetic acid, 1% Triton X-100) with protease inhibitors and phosphatase inhibitors. The cell lysates (1 mg protein) were then pre-cleared with 30 μL of protein A/G-agarose (Santa Cruz, California, USA) at 4 °C for 2 h, and centrifuged at 14,000× *g* at 4 °C for 30 min. Aliquots (50 μL) of the pre-clarified supernatant were saved as input. A total of 250 μL of the pre-cleared supernatant was incubated with the indicated antibody or normal IgG (used as negative control) overnight at 4 °C on a rotary shaker, followed by incubation with protein A/G-agarose for 2 h at 4 °C. After washing twice with IP lysis buffer, the proteins on the beads and input sample were boiled for 10 min in SDS sample loading buffer and separated by SDS-PAGE for Western blotting.

### 4.8. Measurement of Mitochondrial Complex I Activity

The mitochondrial complex I activity was determined by the Complex I Enzyme Activity Microplate Assay Kit (Abcam, Cambridge, UK) according to the manufacturer’s protocol. Briefly, after experimental treatments, mitochondrial complex I was immune-captured within the wells of the microplate, and its activity was determined at 450 nm by following the oxidation of NADH to NAD^+^, and expressed as optical density (OD) value alteration per minute per mg mitochondrial protein (OD_450_/min/mg mitochondrial protein).

### 4.9. Measurement of Mitochondrial ROS Generation

Mitochondrial ROS formation was evaluated by the mitochondria-localizing dye MitoSOX Red, which is oxidized by superoxide, and exhibits red fluorescence. Briefly, after experimental treatments, cells were incubated with 5 μM MitoSOX Red reagent working solution in the dark for 15 min at 37 °C, followed by washing with PBS for three times. Relative fluorescence intensity was qualitatively observed via a phase-contrast fluorescence microscope and quantitatively analyzed with a fluorescent plate reader, at 510 nm excitation and 580 nm emission, and expressed as a percentage of the respective control group value.

### 4.10. Evaluation of Oxidative Stress

Intracellular SOD, CAT, and GPx activity and MDA level were measured as indexes of oxidative stress. The cellular MDA content and antioxidant enzymes (SOD, CAT, and GPx) activities were determine using the corresponding assay kit, respectively, according to the manufacturer’s instructions. The result was normalized to the total protein levels, as measured by the Lowry method using a DC protein assay kit (Bio-Rad, Hercules, CA, USA). 

### 4.11. Assessment of Cell Injury

Cell injury was evaluated by cell viability and lactate dehydrogenase (LDH) release. Cell viability was assessed using the Cell Counting Kit-8 (CCK8, Dojindo, Kumamoto, Japan) method according to manufacturer’s instruction. Briefly, H9c2 cells were plated at a density of 1 × 10^4^ cells/well in 96-well plates. The absorbance was measured at 450 nm with a microplate reader (Bio-Rad Laboratories, Richmond, CA, USA), following treatment with CCK-8 for 2 h at 37 °C, and represented as the percentage of control. The level of LDH release from H9c2 cells was measured using the LDH Cytotoxicity Assay Kit (Beyotime, Beijing, China) according to the manufacturer’s protocol and was presented as a percentage of LDH activity found in the media to the total activity.

### 4.12. Statistical Analysis

All data are presented as means ± SD. Differences among groups were determined by one-way analysis of variance (ANOVA) followed by protected least significant difference Fisher’s test. *p* values <0.05 were considered to indicate statistical significance.
